# Current News

**Published:** 1899-02

**Authors:** 


### Current News.

### SOUTHERN BRANCH OF THE NATIONAL DENTAL ASSOCIATION.

The Southern Branch of the National Dental Association, by invitation of the Louisiana State Dental Society, will hold its second annual meeting in New Orleans, La., February 9, 10, 11, and 13, 1899. The following day is Mardi Gras. Circulars will be issued later giving details as to railroad and hotel rates, etc. All members of the National Dental Association and the American Medical Association are cordially invited as guests of the Southern Branch.

The following is a list of hotels and boarding houses the visiting members of the Southern Branch of the National Dental Association may find convenient and desirable while attending the meeting. It will be necessary to notify proprietors at least ten days in advance to secure good rooms.

### HOTELS.

St. Charles Hotel, American plan, $4.00 per day and up according to location of room. European plan, $2.50 and upward.

Hotel Royal (French quarter), American or European plan, $2.50 to $4.00 per day, according to location of room.

Cosmopolitan Hotel, European plan, from $1.50 upward.

Antoine's Hotel (French), 713-717 St. Louis Street, $2.00 and $3.00.

Hotel Denechaud, American or European plan, from $2.00 to $3.50 per day.

Metropole Hotel, American plan, $2.00 per day.

ROOMS.

Fabacher's Hotel, fifty cents to $1.00 per day.

Penn's Hotel, fifty cents to $1.00 per day.

Commercial Hotel, $1.00 to $2.00 per day.

Metropole Hotel, fifty cents to $1.00 per day.

Rooms (French), 235, 237, 239 Bourbon Street, from $1.00 to $3.00.

PRIVATE FAMILIES.

Mrs. Hawzw, 1350 Magazine Street, rooms with board, $1.00 to $2.00 per day; special rates by the week or month.

Mrs. Joseph B. Davis, 1710 Prytania Street.

Mrs. C. R. Van Wick, 1819 Annunciation Street.

Miss Lulu Bailey, 846 Camp Street.

Any information concerning the above will be cheerfully furnished by the chairman of the Hotels and Quarters Committee, Dr. J. Rollo Knapp, 620 Canal Street, New Orleans, La., or by Dr. Wallace Wood, Jr., 625 Canal Street, New Orleans, La., the secretary of the Executive Committee of the Louisiana State Dental Society.

WILLIAM ERNEST WALKER,
*President Southern Branch National Dental Association.*

---

### ODONTOLOGICAL SOCIETY OF ROCKFORD, ILL.

THE following is the programme for 1898–99 of the Odontological Society of Rockford, Ill., organized November 19, 1897.

December 16, Dr. J. L. Palmer, "Care of Children's Teeth." Discussion to be opened by Dr. E. S. Tebbetts and Dr. C. B. Helm.

January 20, Dr. J. E. Harned, "Anæsthetics and their Use in Dentistry." Discussion to be opened by Dr. H. C. Gill and Dr. M. A. Banks.

February 17, Dr. A. M. Harrison, "A Dentist's Record and Account Books." Discussion to be opened by Dr. C. J. Sowle and Dr. M. R. Harned.

March 17, Dr. C. B. Helm, "The Border-Line between Crowns and Fillings." Discussion to be opened by Dr. C. A. Kitchen and Dr. E. H. Allen.

April 21, Dr. J. J. Reed, "Habits and Conditions of the

Mouth." Discussion to be opened by Dr. B. F. Ells and Dr. A. M. Harrison.

May 19, Dr. E. S. Tebbetts, "Making Dental Instruments." Discussion to be opened by Dr. J. L. Palmer and Dr. J. J. Reed.

June 16, Dr. M. A. Banks, "Sulphuric Acid in the Treatment of Pulpless Teeth." Discussion to be opened by Dr. F. C. Gill and Dr. J. E. Harned.

October 20, Dr. E. H. Allen, "Comparison of Filling-Materials." Discussion to be opened by Dr. Bryant Kerr and Dr. M. L. Hanaford.

November 17, Dr. C. A. Kitchen, "President's Résumé of the Year's Work." Discussion general.

Dr. C. A. Kitchen, *President.*
Dr. C. B. Helm, *Secretary.*

---

### THE NEW YORK INSTITUTE OF STOMATOLOGY.

The annual meeting of The New York Institute of Stomatology was held Tuesday evening, December 6, 1898. The following officers were elected for the year 1899:

President, E. A. Bogue; Vice-President, C. A. Woodward; Recording Secretary, F. Milton Smith; Corresponding Secretary, George A. Wilson; Treasurer, J. A. Bishop; Editor, Frederick L. Bogue; Curator, J. G. Palmer.

*Executive Committee.*—Charles O. Kimball, chairman, S. E. Davenport, J. Morgan Howe.

---

### OHIO STATE DENTAL SOCIETY.

The officers of the Ohio State Dental Society for 1899 are as follows:

President, L. P. Bethel, Kent; First Vice-President, L. L. Barber, Toledo; Second Vice-President, H. F. Harvey, Cleveland; Secretary, S. D. Ruggles, Portsmouth; Treasurer, C. I. Keely, Hamilton.

Yours truly,

S. D. Ruggles,
*Secretary.*

